# UV-B promotes flavonoid biosynthesis in *Ginkgo biloba* by inducing the *GbHY5*-*GbMYB1*-*GbFLS* module

**DOI:** 10.1093/hr/uhad118

**Published:** 2023-06-02

**Authors:** Sian Liu, Xiaoyin Gu, Yanbing Jiang, Lu Wang, Nan Xiao, Yadi Chen, Biao Jin, Li Wang, Weixing Li

**Affiliations:** College of Horticulture and Landscape Architecture, Yangzhou University, Yangzhou 225009, China; College of Horticulture and Landscape Architecture, Yangzhou University, Yangzhou 225009, China; College of Horticulture and Landscape Architecture, Yangzhou University, Yangzhou 225009, China; College of Horticulture and Landscape Architecture, Yangzhou University, Yangzhou 225009, China; College of Horticulture and Landscape Architecture, Yangzhou University, Yangzhou 225009, China; College of Horticulture and Landscape Architecture, Yangzhou University, Yangzhou 225009, China; College of Horticulture and Landscape Architecture, Yangzhou University, Yangzhou 225009, China; College of Horticulture and Landscape Architecture, Yangzhou University, Yangzhou 225009, China; College of Horticulture and Landscape Architecture, Yangzhou University, Yangzhou 225009, China

## Abstract

*Ginkgo biloba* (ginkgo) leaves have medicinal value due to their high levels of secondary metabolites, such as flavonoids. We found that the flavonoid content in ginkgo leaves increases significantly at high altitudes (Qinghai-Tibet Plateau). Considering that high UV-B radiation is among the key environmental characteristics of the Qinghai-Tibet Plateau, we carried out simulated UV-B treatments on ginkgo seedlings and found that the flavonoid content of the leaves increased significantly following the treatments. Combined with results from our previous studies, we determined that the transcription factor GbHY5 may play a key role in responses to UV-B radiation. Overexpression of *GbHY5* significantly promoted the accumulation of flavonoids in both ginkgo callus and *Arabidopsis thaliana*. Furthermore, yeast two-hybrid and real-time quantitative PCR showed that GbHY5 promoted the expression of *GbMYB1* by interacting with GbMYB1 protein. Overexpression of *GbMYB1* in ginkgo callus and *A. thaliana* also significantly promoted flavonoid biosynthesis. *GbFLS* encodes a key enzyme in flavonoid biosynthesis, and its promoter has binding elements of GbHY5 and GbMYB1. A dual-luciferase reporter assay indicated that while GbHY5 and GbMYB1 activated the expression of *GbFLS* individually, their co-expression achieved greater activation. Our analyses reveal the molecular mechanisms by which the UV-B-induced *GbHY5*-*GbMYB1*-*GbFLS* module promotes flavonoid biosynthesis in ginkgo, and they provide insight into the use of UV-B radiation to enhance the flavonoid content of ginkgo leaves.

## Introduction

Ultraviolet (UV)-B radiation (280–315 nm) is not only an important component of sunlight but also an important signaling molecule [1]. UV-B radiation can directly or indirectly affect the growth, development, morphology, and metabolism of plants, and it can significantly affect the biosynthesis of various secondary metabolites [2]. High doses of UV-B radiation typically cause damage to plant leaves, which presents as curling and wilting [3]. In addition, certain doses of UV-B radiation cause dwarfism due to shortened internodes, and higher intensities of UV-B radiation are associated with more pronounced effects on growth [4]. Damage to plants by UV-B radiation is largely attributable to the accumulation of large amounts of reactive oxygen species (ROS), which damage cells and destroy intracellular proteins and DNA [5]. To counteract the damage caused by UV-B, plants increase antioxidant enzyme activity, absorb UV-B, and scavenge ROS via flavonoid accumulation [6]. For example, under UV-B treatment the flavonol content of *Camellia sinensis* leaves increased 1.5- to 2-fold [7, 8] and the flavonoid contents of rice, Tartary buckwheat, and apple increased significantly [9–11].

UV-B affects plant growth and development, as well as the biosynthesis of secondary metabolites, by regulating gene transcription. UV RESISTANCE LOCUS8 (UVR8), a specific receptor for UV-B, is present as a dimer in the cytoplasm in the absence of UV-B, but rapidly depolymerizes into monomers in the nucleus upon exposure to UV-B radiation [12–14]. UV-B-activated UVR8 inhibits the degradation of ELONGATED HYPOCOTYL 5 (HY5) by binding to CONSTITUTIVELY PHOTOMORPHOGENIC 1 (COP1) [15]. HY5, a transcription factor in the basic leucine zipper (bZIP) family, is a key player downstream of COP1 that controls photomorphogenesis and flavonoid biosynthesis [16–18]. In *Arabidopsis thaliana*, HY5 levels are directly correlated with inhibited hypocotyl growth and increased anthocyanin content in seedlings [19]. HY5 promotes anthocyanin accumulation by activating the expression of the anthocyanin regulatory gene *SlAN1*, as well as anthocyanin biosynthesis genes (e.g. *chalcone synthase*, *dihydroflavonol 4-reductase*, and *anthocyanidin synthase*) [20]. In addition, the transcriptional activity of MYB12 and MYB111, key regulators of flavonol biosynthesis, is dependent on HY5 in the presence of both UV-B and visible light [21]. In apple, HY5 can activate the *flavonol synthase* (*FLS*) promoter to increase the flavonoid content, and MYB22 enhances this response [22].

The ancient, ‘living fossil’ plant *Ginkgo biloba* (ginkgo) is not only an ornamental tree but also an important medicinal plant due to high contents of secondary metabolites, such as flavonoids and terpene lactones, in the leaves [23, 24]. Ginkgo leaf extract (GBE) is effective in the treatment of cardiovascular diseases, dementia, and asthma [23]. Among the various compounds that constitute GBE, flavonoids are the most abundant, and the pharmacopoeias of multiple countries stipulate that the flavonoid content must comprise >24% of GBEs [25]. Previous studies have found that various environmental factors can affect flavonoid accumulation in plants, and that UV-B radiation can significantly promote flavonoid biosynthesis [6, 26–28]. We previously found that long-term (7-, 14-, and 21-day) UV-B treatment of ginkgo seedlings can significantly promote flavonoid accumulation [29]. Transcriptome sequencing has revealed that *GbHY5* may be a key gene in promoting flavonoid accumulation in response to UV-B signals [29], but its function and regulatory mechanisms remain unclear.

In this study, we found that the phenotypes of 5-year-old ginkgo trees from low-altitude (LA) Pizhou and high-altitude (HA) Qinghai-Tibet Plateau (Nyingchi) differed significantly, and that ginkgo leaves from HA-grown trees had significantly higher contents of total flavonoids and flavonol glycosides than did leaves from LA-grown trees. Using simulated UV-B treatments, we further confirmed that UV-B radiation is the main factor promoting flavonoid accumulation in ginkgo seedlings. Our results, along with previous transcriptomic data, point to GbHY5 as a key transcription factor that responds to UV-B radiation and promotes flavonoid biosynthesis in ginkgo. Based on transcriptome sequencing, yeast two-hybrid (Y2H) experiments, and dual-luciferase reporter assays, we identified the molecular mechanisms by which the *GbHY5*-*GbMYB1*-*GbFLS* pathway promotes flavonoid biosynthesis in ginkgo in response to UV-B radiation.

## Results

### Phenotype and flavonoid content of ginkgo plants grown at different altitudes

**Figure 1:**
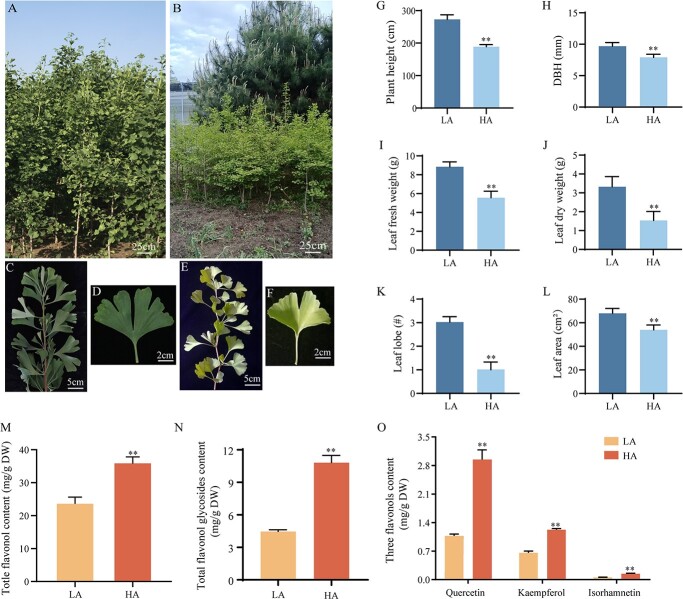
Phenotype and flavonoid content of LA and HA ginkgo. (A, C, D) Phenotypes of LA- and (B, E, and F) HA-grown trees; and (G) plant height (*n* = 10), (H) DBH (*n* = 10), (I) leaf fresh weight, (J) dry leaf weight, (K) number of leaf lobes (*n* = 15), (L) leaf area (*n* = 15), (M) total flavonoids (*n* = 3), (N) total flavonol glycosides (*n* = 3), and (O) quercetin, kaempferol, and isorhamnetin contents of HA- and LA-grown trees (*n* = 3). In (I) and (J) three replicates were set, with 30 leaves each, and the *Y* axis represents the weight per 30 leaves. Asterisks indicate a significant difference as determined by a Student’s *t*-test (*P < .05, **P < .01).

Five-year-old trees from the LA and HA plantations exhibited distinct phenotypes (Fig. 1A–F). Compared with trees growing at LA, the HA trees were smaller and had a smaller diameter at breast height (DBH), leaf fresh weight, leaf dry weight, number of leaf lobes, and leaf area (Fig. 1G–L). However, the total leaf flavonoid content of trees grown at HA was 1.59 times than that of trees grown at LA, and the total content of flavonoid glycosides was 2.4 times higher (Fig. 1M–N). Specifically, the quercetin content of the HA trees was 2.95 mg/g (dry weight), 2.76 times that of the LA trees; the kaempferol content was 1.22 mg/g, 1.85 times that of LA trees, and the isorhamnetin content was 0.14 mg/g, 2.8 times that of LA trees (Fig. 1O).

### Physiological changes and *GbHY5* expression

**Figure 2:**
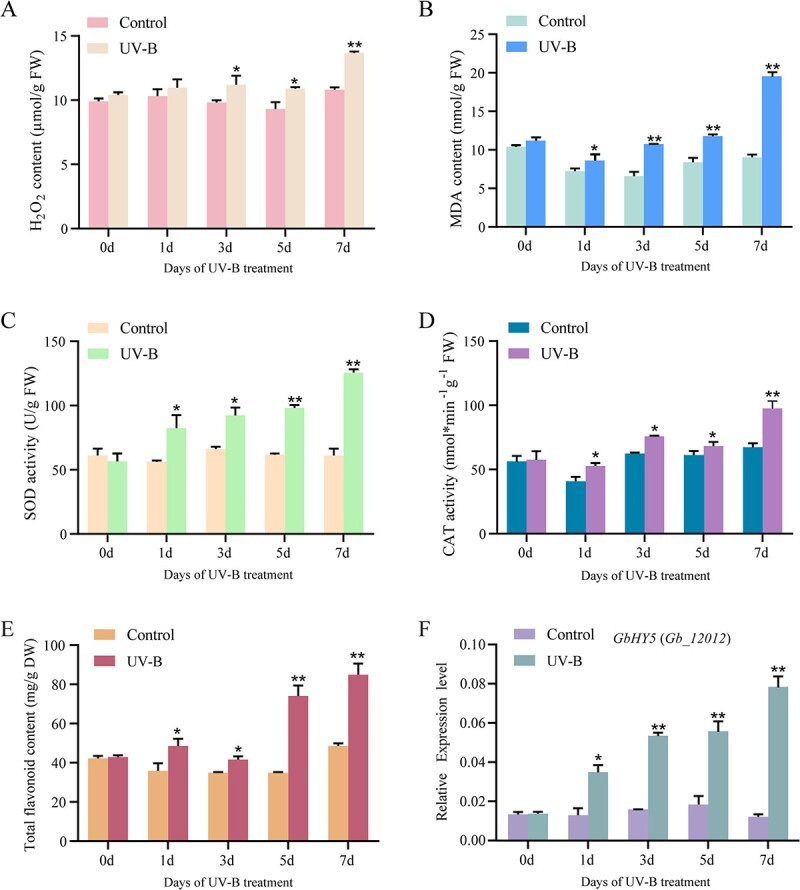
Physiology of ginkgo seedlings following UV-B treatment. (A) H_2_O_2_ content, (B) MDA content, (C) SOD activity, (D) CAT activity, (E) total flavonoid content, and (F) *GbHY5* expression level. Asterisks indicate a significant difference as determined by a Student’s *t*-test (*P < .05, **P < .01). The error bars indicate the mean ± standard deviation (*n* = 3).

Intense UV-B radiation is a key characteristic of HA regions: the average daily dose of UV-B in Nyingchi can be as high as 7.14 kJ/m^2^/day, far exceeding that of lowland areas [29]. Studies have found that moderate UV-B radiation promotes flavonoid accumulation in plants [7]. To explore whether the high flavonoid content observed in the leaves of HA trees is related to strong UV-B radiation, we subjected ginkgo seedlings to treatments simulating the average UV-B dose in Nyingchi. The malondialdehyde (MDA) and hydrogen peroxide (H_2_O_2_) contents exhibited similar trends in response to the treatment; both increased slightly between days 1 and 5, then increased significantly as of day 7 (Fig. 2A and B). The activity of the ROS scavengers superoxide dismutase (SOD) and catalase (CAT) increased as the treatment period progressed (Fig. 2C and D). Flavonoids may also scavenge ROS under stress, and the total flavonoid content of the leaves increased significantly following treatment (Fig. 2E). We previously used RNA sequencing (RNA-seq) to demonstrate that the expression of *GbHY5* in ginkgo leaves increases significantly under long-term UV-B treatment (14 days), implying that this gene is important in responses to UV-B [29]. Here, we used real-time quantitative reverse transcription–PCR (qRT–PCR) to demonstrate that the expression of *GbHY5* increased significantly as the duration of the treatment increased, and that trends in its expression corresponded to trends in total flavonoid accumulation (Fig. 2F).

### *GbHY5* promotes flavonoid accumulation

**Figure 3:**
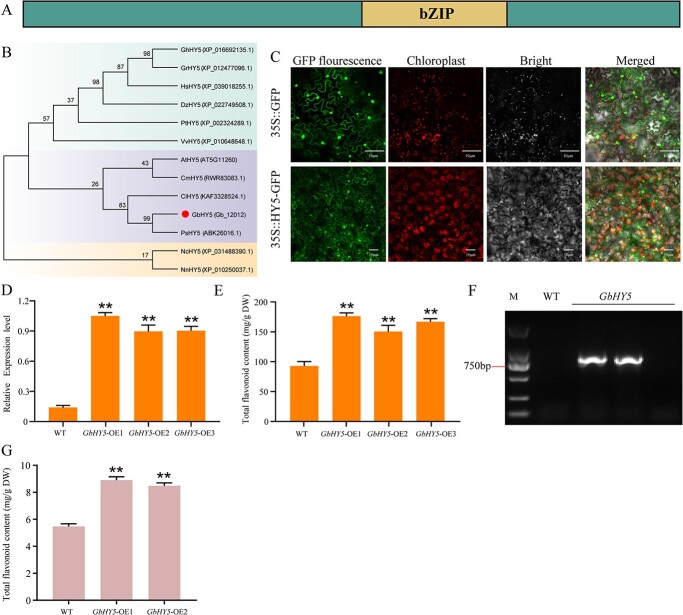
*GbHY5* promotes flavonoid accumulation. (A) Protein structure and (B) phylogenetic tree. Cm, *Cinnamomum micranthum* f. *kanehirae*; Nc, *Nymphaea colorata*; Gh, *Gossypium hirsutum*; Hs, *Hibiscus syriacus*; Cl, *Carex littledalei*; Nn, *Nelumbo nucifera*; Dz, *Durio zibethinus*; Gr, *Gossypium raimondii*; Pt, *Populus trichocarpa*; Vv, *Vitis vinifera*; At, *Arabidopsis thaliana*; Ps, *Picea sitchensis*; Gb, *Ginkgo biloba*. (C) Subcellular localization of GbHY5; and (D) *GbHY5* expression level, (E) total flavonoid content, (F) positive detection, and (G) total flavonoid content of *GbHY5-*overexpressing *A. thaliana* plants. Asterisks indicate a significant difference as determined by a Student’s *t*-test (*P < .05, **P < .01). The error bars indicate the mean ± standard deviation (*n* = 3).

GbHY5 belongs to the bZIP transcription factor family and has a typical bZIP domain (Fig. 3A). Phylogenetic analyses have demonstrated that GbHY5 protein is in a clade that also includes PsHY5 of *Picea sitchensis* (Fig. 3B). Following the transformation of 35S::GbHY5-GFP into *Nicotiana benthamiana* leaves, green fluorescent protein (GFP) fluorescence was observed in the nucleus, cell membrane, and cytoplasm, indicating the localization of GbHY5 to the nucleus, cell membrane, and cytoplasm (Fig. 3C). To explore the function of *GbHY5*, we transformed 35S::GbHY5-GFP into ginkgo calli. qRT–PCR indicated that the expression of *GbHY5* increased significantly in all three callus lines (Fig. 3D). The total flavonoid content in the transgenic calli increased by 1.5–1.75 times compared with wild-type (WT) plants (Fig. 3E). In addition, we transformed 35S::GbHY5-GFP into *A. thaliana*, obtaining two transgenic lines (Fig. 3F). The transgenic plants exhibited no obvious phenotypic differences compared with WT plants (Fig. 3G and H), but the total flavonoid content increased significantly (Fig. 3I). These data indicate that *GbHY5* promotes flavonoid accumulation in plants.

### Identification of genes downstream of *GbHY5*

**Figure 4:**
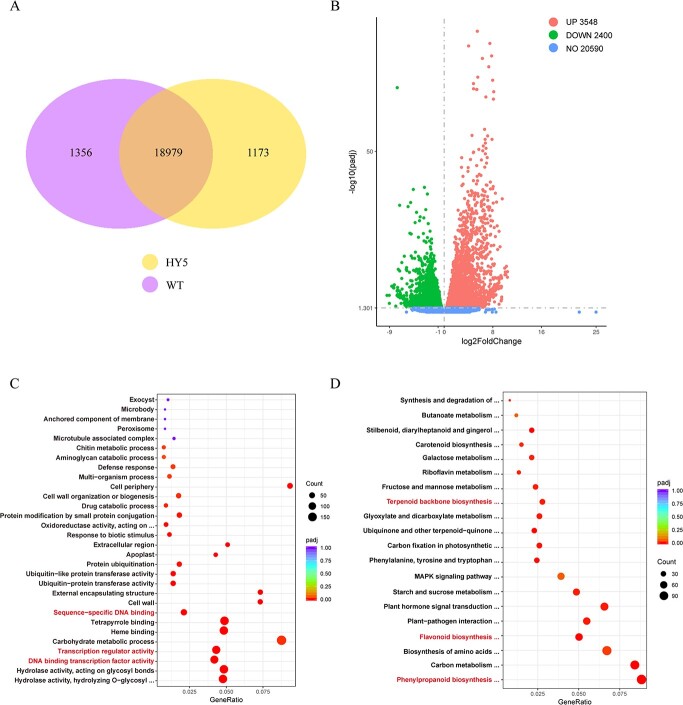
Enrichment analysis of differentially expressed genes. (A) Genes specifically expressed in WT and *GbHY5*-overexpressing calli. (B) Volcano plots of the differentially expressed genes. Red dots represent upregulated expressed genes, green dots represent downregulated expressed genes, and blue dots represent genes with no significant difference. (C) GO enrichment analysis results. (D) KEGG enrichment analysis results.

We conducted RNA-seq of *GbHY5*-overexpressing transgenic ginkgo calli to explore the mechanisms by which GbHY5 regulates flavonoid accumulation in ginkgo. In total, 39.54 Gb of clean bases were obtained following the removal of adapters, N-containing bases, and reads with low sequence quality (Supplementary Data Table S1). The clean reads were mapped to the ginkgo genome, and the total mapped rates of the six samples were all >94% (Supplementary Data Table S2). A total of 21 508 expressed genes were obtained by RNA-seq, of which 1173 were specifically expressed in the transgenic calli (Fig. 4A). Differential expression analysis identified a total of 5948 differentially expressed genes (DEGs), of which 2400 were upregulated and 3548 were downregulated (Fig. 4B). Gene Ontology (GO) enrichment analysis of the DEGs indicated that they were enriched in multiple GO terms related to transcriptional regulation, including ‘transcription regulator activity’, ‘DNA binding transcription factor activity’, and ‘sequence-specific DNA binding’ (Fig. 4C). Kyoto Encyclopedia of Genes and Genomes (KEGG) enrichment analysis indicated that the DEGs were enriched in multiple secondary metabolic biosynthesis pathways, including flavonoid biosynthesis, phenylpropanoid biosynthesis, and terpenoid backbone biosynthesis (Fig. 4D).

**Figure 5:**
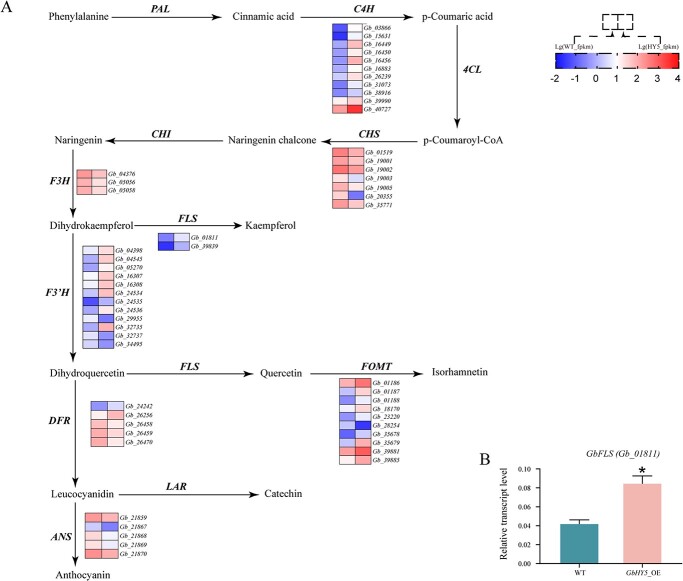
DEGs in ginkgo flavonoid biosynthesis pathway. (A) DEGs in the flavonoid biosynthesis pathway, and (B) qRT–PCR detection of the expression level of *GbFLS* in *GbHY5*-overexpressing callus. Asterisks indicate a significant difference as determined by a Student’s *t*-test (*P < .05, **P < .01). The error bars indicate the mean ± standard deviation (*n* = 3).

Because *GbHY5* significantly promotes flavonoid accumulation, we focused on the flavonoid biosynthesis pathway. We found that 55 DEGs were involved in this pathway, including 11 *cinnamate 4-hydroxylase* (*C4H*) genes, 7 *chalcone synthase* (*CHS*) genes, 3 *flavonoid 3-hydroxylase* (*F3H*) genes, 2 *flavonol synthase* (*FLS*) genes, 12 *flavonoid 3′-hydroxylase* (*F3′H*) genes, 5 *dihydroflavonol 4-reductase*and (*DFR*) genes, 5 *anthocyanidin synthase* (*ANS*) genes, and 10 *flavonoid o-methyltransferase* (*FOMT*) genes (Fig. 5A). Most of the *C4H*, *FLS*, *F3′H*, and *FOMT* genes were upregulated (Fig. 5A). In addition, we also found that most of the genes involved in anthocyanin biosynthesis (*DFR*, *LAR*) were downregulated (Fig. 5A). *FLS* encodes the main biosynthetic enzyme for flavonols such as kaempferol and quercetin. We performed qRT–PCR on the most significantly upregulated *GbFLS* gene, *Gb_08111*, to confirm that its expression levels increased significantly following *GbHY5* overexpression (Fig. 5B).

### GbMYB1 protein interacts with GbHY5 and promotes flavonoid biosynthesis

**Figure 6:**
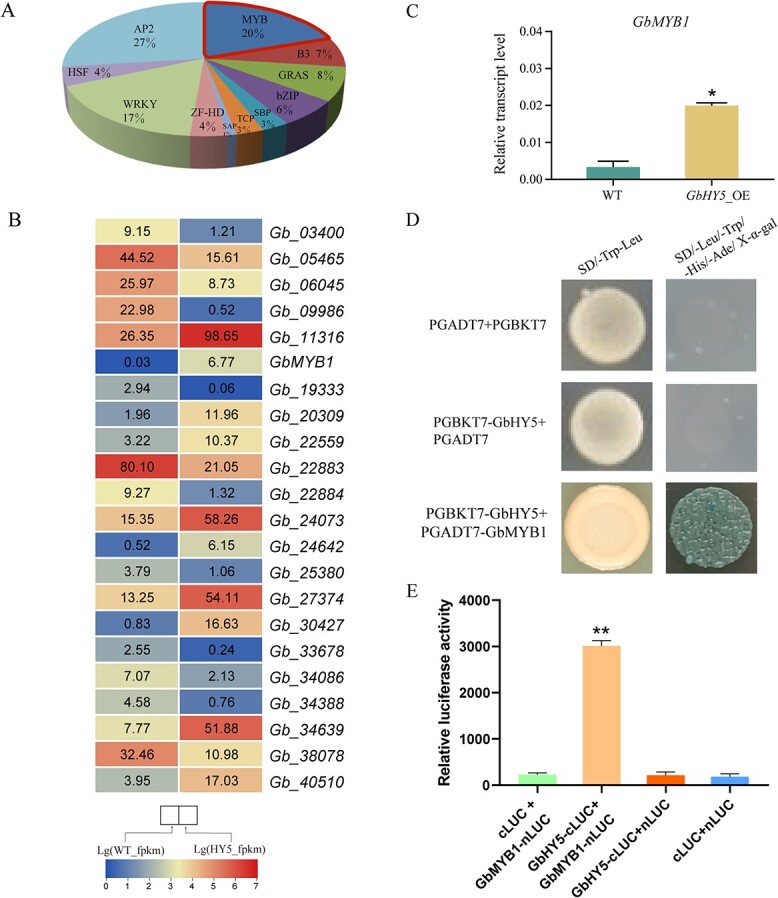
GbHY5 protein interacts with GbMYB1. (A) Type and percentage of differentially expressed transcription factors and (B) differentially expressed MYB transcription factors. Numbers represent the FPKM (fragments per kilobase of transcript per million mapped reads) values. (C) Expression level of *GbMYB1* in *GbHY5*-overexpressing callus, and (D) Y2H assay of interactions between GbHY5 and GbMYB1. (E) Split-luciferase complementation assays showing the interactions between GbHY5 and GbMYB1. Asterisks indicate a significant difference as determined by a Student’s *t*-test (*P < .05, **P < .01). The error bars indicate the mean ± standard deviation (*n* = 3).

Transcription factors, particularly MYB, play important roles in the regulation of plant flavonoid biosynthesis. A total of 111 differentially expressed transcription factors were identified via transcriptome analysis; these were classified into 11 families, among which AP2 and MYB were the most abundant (Fig. 6A). Twenty-two members of the MYB family were differentially expressed; half were upregulated and the other half downregulated (Fig. 6B). Of these genes, *GbMYB1* had the largest fold upregulation (Fig. 6B), and qRT–PCR indicated that its expression increased significantly following the overexpression of *GbHY5* (Fig. 6C). Previous studies have demonstrated that ZbHY5 protein interacts with ZbMYB113 in *Zanthoxylum bungeanum* [30]. To test whether GbHY5 interacts with GbMYB1, we constructed pGBKT7-GbHY5 and pGADT7-GbMYB1 vectors, and, using a Y2H experiment, confirmed the hypothesized interaction (Fig. 6D). Furthermore, split-luciferase (split-LUC) complementation assays further demonstrated the interactions between GbHY5 and GbMYB1 (Fig. 6E).

**Figure 7:**
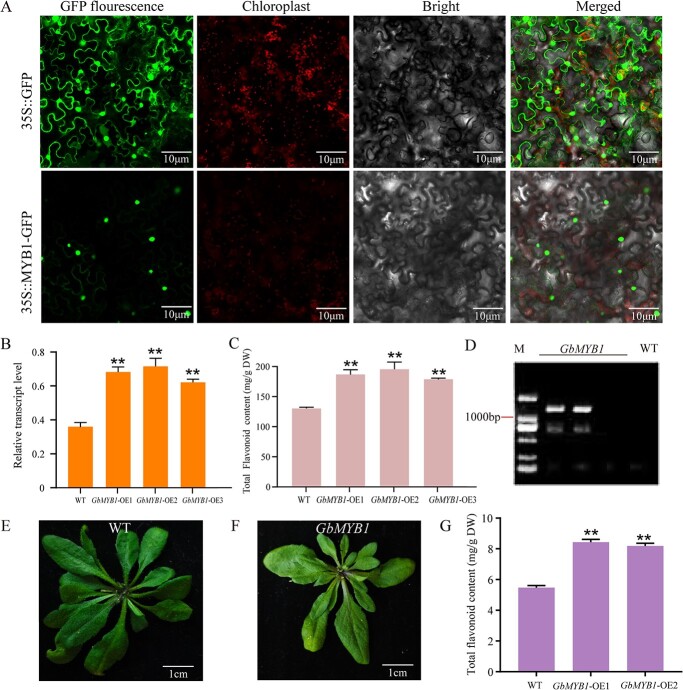
GbMYB1 positively regulates flavonoid biosynthesis. (A) Subcellular localization of GbMYB1, (B) *GbMYB1* expression level, and (C) total flavonoid content in *GbMYB1*-overexpressing ginkgo callus; and (D) the positive detection, (E and F) phenotype, and (G) total flavonoid content of *GbMYB1*-overexpressing *A. thaliana*. Asterisks indicate a significant difference as determined by a Student’s *t*-test (*P < .05, **P < .01). The error bars indicate the mean ± standard deviation (*n* = 3).

We isolated *GbMYB1*, which has a coding sequence (CDS) of 1326 bp and encodes 441 amino acids. Phylogenetic analysis of *GbMYB1* with *Arabidopsis MYBs* revealed that *GbMYB1* with *AtMYB1* and *AtMYB109* belong to Subgroup 23 (SG 23) *MYB* (Supplementary Data Fig. S1). Further phylogenetic analysis of *GbMYB1* with *MYB1* from other plants showed *GbMYB1* in one clade with *PpMYB1* of *Physcomitrium patens* (Supplementary Data Fig. S2). Phylogenetic tree analysis showed that the *MYB1* genes from different plant species can be classified into three clades, and that *GbMYB1* is in the same clade as *PpMYB1* of *P. patens* (Supplementary Data Fig. S2). A 35S::GbMYB1-GFP vector was constructed and transferred into the leaves of *N. benthamiana* for subcellular localization experiments, and the results indicated that GbMYB1 is localized in the nucleus (Fig. 7A). To explore the function of *GbMYB1*, the vector was also transferred into ginkgo calli, and the expression of *GbMYB1* increased in the three callus lines (Fig. 7B). Subsequently, the total flavonoid content of *GbMYB1*-overexpressing callus was measured, and was found to be 1.42–1.51 times that of WT plants (Fig. 7C). In addition, we transformed the 35S::GbMYB1-GFP vector into *A. thaliana* and obtained two transgenic lines (Fig. 7D). While the phenotype of the transgenic plants did not change significantly, the total flavonoid content significantly increased (Fig. 7E–G). These results suggest that *GbMYB1* promotes flavonoid accumulation.

### GbHY5 and GbMYB1 activate *GbFLS* promoter activity

**Figure 8:**
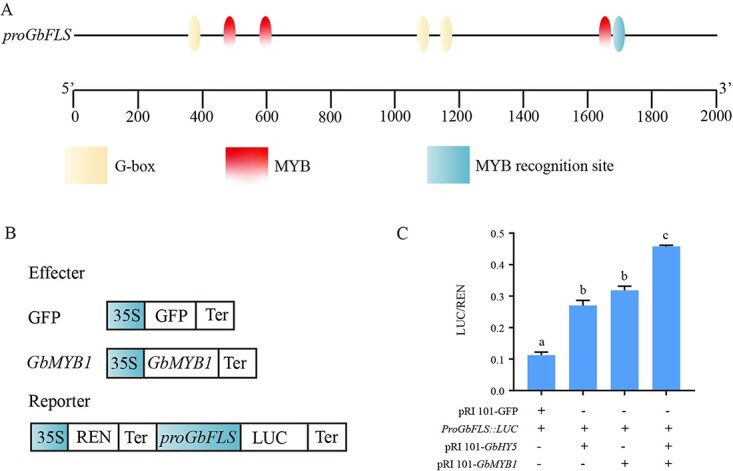
GbHY5 and GbMYB1 activate the promoter activity of *GbFLS*. (A) Promoter element analysis of *GbFLS*. Different colored squares represent different motifs. (B) Dual-luciferase vector construction, and (C) the dual-luciferase reporter assay. Different letters within each treatment indicate significant differences at *P* ≤ .05 (one-way ANOVA followed by Dunnett’s test). The error bars indicate the mean ± standard deviation (*n* = 3).

*GbHY5*, *GbMYB1*, and *GbFLS* all promote flavonoid accumulation in ginkgo; however, the regulatory relationship between them remains unclear. To investigate whether GbHY5 and GbMYB1 regulate *GbFLS*, we cloned the 1937-bp promoter sequence of *GbFLS* and analyzed it for *cis*-acting elements. PlantCARE analysis indicated that the *GbFLS* promoter has a potential G-box binding element for HY5 and a potential MYB binding element/MYB recognition site for MYB (Fig. 8A). To explore whether GbHY5 and GbMYB1 regulate the expression of *GbFLS* by binding the *cis*-acting element in the *GbFLS* promoter, we cloned the *GbFLS* promoter into the vector pGreen II 0800-LUC and conducted a dual-luciferase reporter assay (Fig. 8B). Compared with the control, GbHY5 promoted a 1.41-fold increase in *GbFLS* promoter activity, whereas GbMYB1 promoted a 1.84-fold increase (Fig. 8C). Interestingly, *GbFLS* promoter activity increased 3.44-fold when GbHY5 and GbMYB1 were co-expressed (Fig. 8C). These results indicate that while GbHY5 and GbMYB1 can individually enhance the activity of the *GbFLS* promoter, their co-expression leads to greater enhancement (Fig. 8C).

## Discussion

Increased UV-B radiation leads to phenotypic changes and increased accumulation of secondary metabolites in plants. Previous studies have found that UV-B radiation can reduce the leaf area in rice, sunflower, and lettuce, and inhibit leaf growth in *A. thaliana* [31, 32]. In cucumber, UV-B radiation not only causes a significant reduction in leaf area but also results in decreased plant height, petiole length, and biomass [4]. UV-B radiation can also lead to shortened internodes, reductions in leaf number, leaf curling, and increased axillary branches [33, 34]. In addition, the flavonoid content increases significantly under UV-B radiation to prevent damage. For example, *C. sinensis*, *Caryopteris mongolica*, *Cymbopogon citratus*, and *Withania somnifera* exhibit significant increases in flavonoid content when exposed to UV-B radiation [7, 35–37]. Strong UV-B radiation is a key characteristic of HA regions, and HA plants exhibit phenotypes and flavonoid accumulation patterns similar to those of plants subjected to UV-B treatment. Maca, which grows in the Andes, adapts to the environment by changing leaf morphogenesis and reducing the leaf area [38]. Compared with *A. thaliana* ecotype Columbia, the HA ecotype Tibet-0, which grows on the Qinghai-Tibet Plateau, has fewer leaves, more branches, and a smaller stature [39]. Peach, apple, and Tartary buckwheat grown at HA exhibit strong flavonoid accumulation [10, 11, 40, 41]. We found that the plant height, DBH, leaf fresh and dry weights, and leaf area of ginkgo grown at HA were significantly lower than those of plants grown at LA. We also found that the contents of flavonols such as kaempferol, quercetin, and isorhamnetin, as well as the total flavonol glycoside and flavonoid content in the leaves of HA trees were significantly higher than those of LA trees. Our UV-B simulation experiments on ginkgo seedlings indicate that ROS and damage to the membrane system increased as treatment time increased. The scavenging activities of enzymes such as SOD and CAT increased significantly to alleviate the damage caused by excessive ROS, as did the flavonoid content.

*HY5* plays an important role in regulating plant root growth, biosynthesis and accumulation of secondary metabolites, cold tolerance, and responses to various hormonal signals [42–45]. The hypocotyls of *A. thaliana hy5* mutants are significantly longer than those of WT plants when grown under both light and dark conditions [46, 47], and they have more lateral roots and root hairs [48]. In addition to inhibiting hypocotyl growth, *HY5* plays an important role in regulating the accumulation of secondary metabolites in response to light signals. In *Artemisia annua AaHY5* indirectly controls artemisinin synthesis in response to light conditions, and in *C. sinensis CsHY5* mediates UV-B light signaling to promote flavonoid accumulation [49]. The expression of *GbHY5* in ginkgo seedlings increases significantly under long-term UV-B treatment (1–4 weeks) [29]. Our analyses demonstrate that *GbHY5* also responds strongly to UV-B light signals after 1–7 days of UV-B treatment, and that its expression increases over time. HY5 is localized to the nucleus in both *A. thaliana* and *C. sinensis* [49, 50], whereas GbHY5 is present in both the nucleus and cell membrane in ginkgo. Similar to other studies of *HY5* in angiosperms, our experiments using both ginkgo calli and ectopically transformed *A. thaliana* demonstrate that *GbHY5* positively regulates flavonoid accumulation. However, no clear phenotypic changes were observed in transgenic plants, likely because *HY5* differs functionally in some respects between gymnosperms and angiosperms.

**Figure 9:**
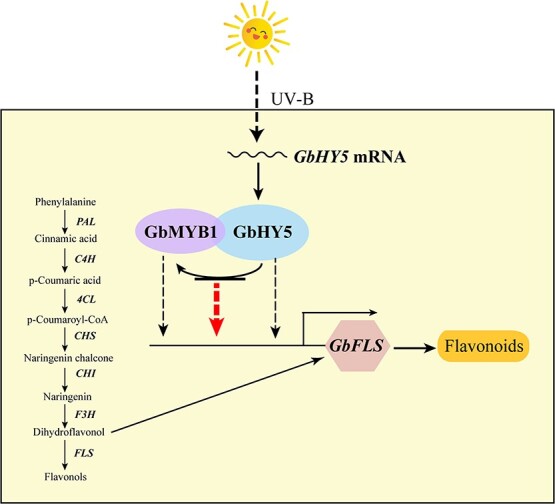
A working model of the proposed *GbHY5*-*GbMYB1*-*GbFLS* module for promoting flavonoid biosynthesis.

Previous studies have found that multiple MYB transcription factors are involved in the regulation of plant flavonoid biosynthesis, including SG7 MYB (AtMYB11, AtMYB12, and AtMYB111), which regulate flavonol biosynthesis in *A. thaliana*, and AtPAP1, which controls anthocyanin biosynthesis [51, 52]. UV-B treatment of blueberry during the green fruit stage increases the expression of *HY5*, thereby upregulating *VcMYBPA1*, downregulating *VcMYBC2*, and promoting the accumulation of anthocyanins [53]. Conversely, an *HY5*-independent pathway in ripe fruit suppresses excessive anthocyanin biosynthesis when exposed to UV-B light by upregulating *VcMYBC2* [53]. In pear, *PyHY5*, alone or in conjunction with *PyBBX18*, promotes the expression of *PyMYB10* and *PyWD40*, leading to anthocyanin biosynthesis [54, 55]. These results suggest that UV-B radiation, *HY5*, and *MYB* have complex and interrelated roles in the regulation of flavonoid biosynthesis. Phylogenetic tree analysis indicated that *GbMYB1*, *AtMYB1*, and *AtMYB109* belong to SG23 MYB. Previous studies reported that SG23 MYB plays an important role in plant stress response [56]. For example, *AtMYB1* responds to salt stress and participates in its tolerance in *Arabidopsis* by regulating ABA [57]. *VdMYB1* positively regulates grape defense response by activating *stilbene synthase gene 2* [58]. The *SlMYB75* gene promotes anthocyanin accumulation in tomato as well as volatile aroma production and can enhance resistance to *Botrytis cinerea* [59, 60]. *SsMYB113* can regulate the accumulation of *Schima superba* flavonoids and improve drought stress tolerance [61]. *AtMYB109* negatively regulates stomatal closure in *Arabidopsis* under osmotic stress [62]. However, our genetic transformation experiments confirmed that *GbMYB1* is a positive regulator of flavonoid biosynthesis in the nucleus. The functional difference between *GbMYB1* and angiosperm SG23 MYB genes may be due to its relatively distant evolutionary relationship with angiosperm *MYB1*. Furthermore, our transcriptome sequencing, qRT–PCR, and Y2H and split-LUC assays demonstrate that *GbHY5* interacts with GbMYB1 and promotes the expression of *GbMYB1*. Previous studies showed that UV-B irradiation promoted the accumulation of total flavonoids but decreased the content of anthocyanins [63], and the expression of all *GbDRF* genes decreased significantly under UV-B irradiation, but the molecular mechanism is unclear [64]. In this study, we found that *GbHY5* promoted most flavonoid synthesis-related genes but inhibited the expression of anthocyanin-related genes such as *DRF* and *ANS*, which initially explained the decrease in anthocyanin content under UV-B irradiation. We found that *GbHY5* was significantly upregulated upon UV-B irradiation and interacted with the GbMYB1 protein. Based on previous reports [65, 66] and our dual luciferase experimental results, it is speculated that GbMYB1 and GbHY5 may be able to promote *GbFLS* expression either by enhancing the activity of the *GbFLS* promoter alone or to co-regulate *GbFLS* through protein interaction (Fig. 9). In conclusion, *GbHY5*-*GbMYB1*-*GbFLS* is an important module for promoting the flavonoid synthesis of ginkgo in response to UV-B irradiation.

## Materials and methods

### Plant materials, growing conditions, and UV-B treatment

Nyingchi (Tibet, China; 34°35′ N, 117°59′ E) is located in the Qinghai-Tibet Plateau and has an average elevation of 3000 m above sea level (asl), whereas Pizhou (Jiangsu, China; 29°67′ N, 94°35′ E) is located on a plain and has an average elevation of 22 m asl. We introduced individuals of *G. biloba* ‘Fozhi’ from LA Pizhou to HA Nyingchi to establish HA ginkgo plantations. The ginkgo seedlings in both places are 5 years old, and the distance between two seedlings is ~50–60 cm. When sampling, we chose ginkgo trees far away from other tree species around the ginkgo plantation. Seedlings of *G. biloba* ‘Fozhi’ were grown in a climate chamber at a temperature of 25°C/18°C (day/night), with a 16-hour light/8-hour dark photoperiod. Following previously described methods [29], 4-month-old seedlings were subjected to low doses of UV-B (7.14 kJ/m^2^/day) for 1, 3, 5, or 7 days, with a 16-hour light (UV-B)/8-hour dark photoperiod. A control group was grown under white light. *Arabidopsis thaliana* ecotype Columbia and *N. benthamiana* were also grown in a climate chamber at a temperature of 23°C/18°C (day/night), with a 16-hour light/8-hour dark photoperiod.

### Gene cloning and vector construction

The CDSs of *GbHY5* and *GbMYB1* were amplified using an LA Taq Kit (Takara Bio Inc., Shiga, Japan) following the manufacturer’s protocols and using ginkgo cDNA as a template. The specific primer sequences are shown in Supplementary Data Table S3. The PCR products were subjected to agarose gel electrophoresis and purified using an AxyPrep DNA Gel Extraction Kit (Axygen Scientific, Union City, CA, USA). The CDSs of *GbHY5* and *GbMYB1* were cloned into the overexpression vector pRI101-GFP using a ClonExpress II One Step Cloning Kit (Vazyme Biotech, Nanjing, China). The same methods were used to clone *GbHY5* into the pGBKT7 vector, and *GbMYB1* into the pGADT7 vector. The CDS of *GbHY5* was cloned into the pCAMBIA1300-cLuc vector, and the CDS of *GbMYB1* was cloned into the pCAMBIA1300-nLuc vector. We used a DNeasy Plant Mini Kit (Qiagen, Hilden, Germany) to extract DNA from the leaves. The DNA was used as a template, and the *GbFLS* promoter was isolated using specific primers and cloned into the vector pGreen II 0800-LUC (Supplementary Data Table S3).

### Promoter element analysis and phylogenetic tree construction

The *cis*-acting elements in the *GbMYB1* promoter were predicted with PlantCARE (http://bioinformatics.psb.ugent.be/webtools/plantcare/html/) and visualized using TBtools [67]. Protein domains were predicted using Pfam (http://pfam.xfam.org/). A multiple sequence alignment was conducted in Clustal X2.1, and phylogenetic trees were constructed in MEGA 11 using the neighbor-joining method and a bootstrap value of 1000 [68].

### Subcellular localization assay

The 35S::GbHY5-GFP and 35S::GbMYB1-GFP vectors were transformed into *Agrobacterium tumefaciens* strain GV3101 and cultured to OD_600_ = 1.0. Following centrifugation, the strain was resuspended in a solution of 10 mM MgCl_2_, 10 mM 2-(N-morpholino) ethanesulfonic acid, and 150 mM acetosyringone. The resuspended solution was then injected into the leaves of 4-week-old *N. benthamiana* plants, and GFP fluorescence was observed using a confocal microscope (LSM880; Zeiss, Oberkochen, Germany) after 2 days of culture.

### Genetic transformation of ginkgo calli and *A. thaliana*

We transformed the 35S::GbHY5-GFP and 35S:: GbMYB1-GFP vectors into *A. tumefaciens* strain EHA105 and infected ginkgo calli following previously described methods [69]. After four days of culture, samples were collected for use in subsequent experiments. We also transformed the two vectors into *A. tumefaciens* strain GV3101 and transformed *A. thaliana* using the floral dip method. Seeds of transformed *A. thaliana* plants were harvested and sown on medium containing kanamycin, and the seedlings were transplanted into pots. PCR was used to assess the success of gene insertion. Screened, *T*_3_-generation positive transgenic plants were used for phenotypic observation and total flavonoid content measurement. The total flavonoid content was determined using a plant flavonoid extraction kit (Suzhou Keming Biotechnology Co., Ltd., Suzhou, China).

### RNA-seq and analysis

Total RNA was extracted from six ginkgo callus samples using an RNAprep Pure Plant Kit (Tiangen, Beijing, China). cDNA synthesis was conducted using an EasyScript First Strand cDNA Synthesis SuperMix Kit (TransGen Biotech Co. Ltd., Beijing, China). The cDNA library was constructed as described previously [69], and RNA-seq was conducted using an Illumina HiSeq 4000 platform (Illumina Inc., San Diego, CA, USA). Clean reads were mapped to the reference genome using HISAT2 [70]. StringTie was used for novel gene prediction, and featureCounts was used to calculate the read count of each gene [70]. Differential expression was analyzed using DESeq2 [71]. Thresholds for significant differential expression were as follows: adjusted *P*-value of <.5 and |log2foldchange| of >1. GO and KEGG enrichment analyses of the DEGs were conducted using clusterProfiler 4.0 [72].

### qRT–PCR

Total RNA was extracted using an RNAprep Pure Plant Kit (Tiangen), following the manufacturer’s protocols. *GAPDH* was used as an internal reference gene [73], and Primer Premier 5.0 was used to design primers for the target gene (Supplementary Data Table S3). qRT–PCR was performed on a CFX96™ Real-Time System (Bio-Rad, Hercules, CA, USA) using a PerfectStart Green qPCR SuperMix Kit (TransGen Biotech Co. Ltd.). Relative gene expression was calculated using 2^−ΔΔCt^ [74].

### Yeast two-hybrid assay

The assay was conducted using a Y2H System (Clontech Laboratories, Mountain View, CA, USA). Yeast suspensions of different combinations (pGBKT7-GbHY5/pGADT7-GbMYB1, pGBKT7-GbHY5/PGADT7) were placed on SD/−Trp/−Leu and SD/−Leu/−Trp/-His/−Ade/X-α-gal media for self-activation activity assays. pGBKT7-GbHY5 and pGADT7-GbMYB1 were co-transfected into Y2H Gold yeast competent cells, cultured on SD/−Trp/−Leu medium for 3 days, and then transferred to SD/−Leu/−Trp/−His/−Ade/X-α-gal medium.

### Dual-luciferase reporter assay and split-luciferase assay

Protoplasts from 1-month-old poplar leaves were isolated following previously described methods [75]. Briefly, 1-month-old poplar leaves were cut into filaments and then placed into an enzyme solution configured with 2% cellulase and 0.5% pectinase and kept in at 28°C in darkness for 3 hours. The liquid was then subjected to centrifugation and 2 mM MES at pH 5.7, 154 mM NaCl, 125 mM CaCl2,5 mM KCl (W5) was added to dilute the poplar leaf protoplasts. Different combinations of plasmids (pRI101-GFP/ProGbFLS::LUC, ProGbFLS::LUC/35S::GbHY5, ProGbFLS::LUC/35S::GbMYB1, ProGbFLS::LUC/35S::GbHY5/35S::GbMYB1, 35S::nLUC/35S::GbMYB1-nLUC and 35S::GbMYB1-nLUC/35S::GbHY5-nLUC) were co-transformed into the protoplasts and cultured for 16 hours. LUC and REN activity was detected using a GLO-MAX20/20 Fluorescence Detector with a Dual Luciferase Reporter Assay Kit (Promega Corp., Madison, WI, USA).

### Statistical analysis

All data presented in this study were collected from more than three independent replicates and were statistically analyzed by Student’s *t*-test or one-way ANOVA.

## Supplementary Material

Web_Material_uhad118

## Data Availability

The sequencing data are available at the NCBI SRA database under accession number PRJNA960813.
