# Supplementary material for: UV-B promotes flavonoid biosynthesis in Ginkgo biloba by inducing the GbHY5-GbMYB1-GbFLS module
Source: Hortic Res. 2023 Jun 2;10(8):uhad118. doi: 10.1093/hr/uhad118 (PMC10402656; doi:10.1093/hr/uhad118)
Supplement: Web_Material_uhad118 [file web_material_uhad118.docx]

UV-B promotes flavonoid biosynthesis in *Ginkgo biloba* by inducing the *GbHY5*-*GbMYB1*-*GbFLS* module

Sian Liu^1^, Xiaoyin Gu^1^, Yanbing Jiang^1^, Lu Wang^1^, Nan Xiao^1^, Yadi Chen^1^, Biao Jin^1^, Li Wang^1*^, Weixing Li^1 *^

^1^ College of Horticulture and Landscape Architecture, Yangzhou University, Yangzhou 225009, China

^*^Corresponding author: liwx@yzu.edu.cn; liwang@yzu.edu.cn


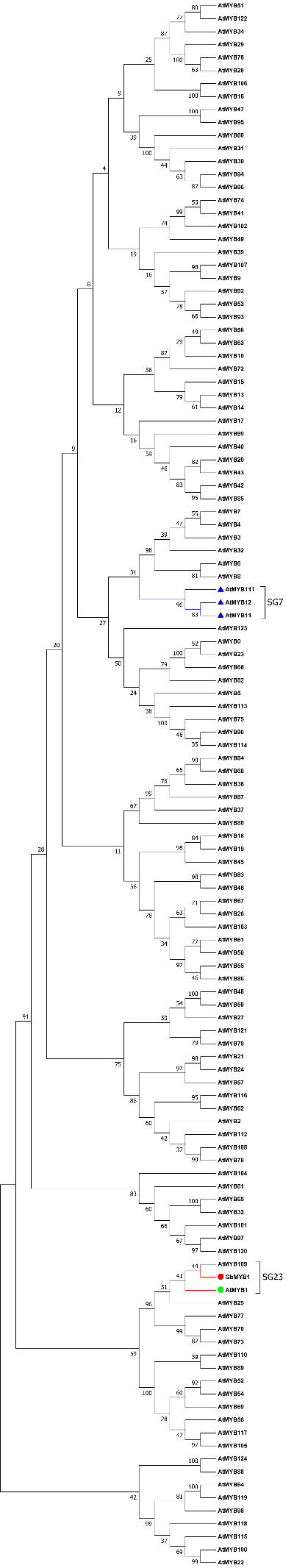


Fig. S1 Phylogenetic tree of *GbMYB1* and *Arabidopsis* *MYBs*


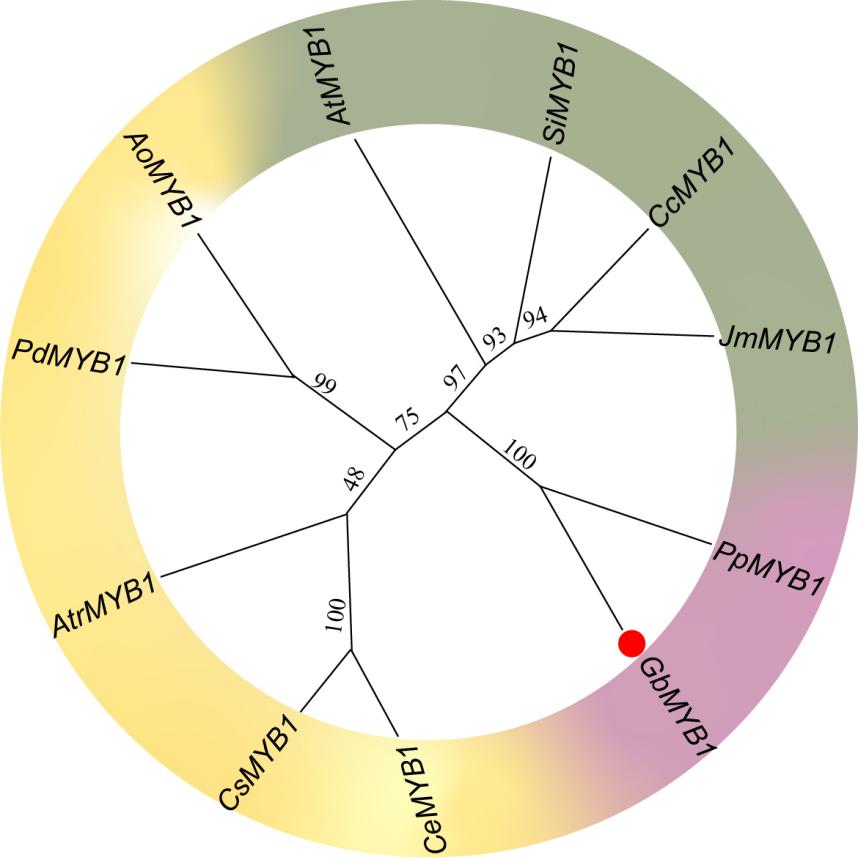


Fig. S2 Phylogenetic tree of *MYB1*

Table S1 Summary of sequencing data quality

| Sample name | Raw reads | Clean reads | clean bases | Error rate (%) | Q20 (%) | Q30 (%) | GC content (%) |
| --- | --- | --- | --- | --- | --- | --- | --- |
| CK-1 | 44175530 | 43285066 | 6.49G | 0.03 | 97.64 | 93.35 | 44.06 |
| CK-2 | 46915714 | 45889806 | 6.88G | 0.03 | 97.58 | 93.28 | 44.11 |
| CK-3 | 44287300 | 43410908 | 6.51G | 0.03 | 97.52 | 93.09 | 43.57 |
| HY5-1 | 43282772 | 42340760 | 6.35G | 0.03 | 97.76 | 93.70 | 43.58 |
| HY5-2 | 45026058 | 44034966 | 6.61G | 0.03 | 97.78 | 93.69 | 45.26 |
| HY5-3 | 45594938 | 44636280 | 6.70G | 0.03 | 97.91 | 94.00 | 44.08 |

Table S2 The result of total reads mapped to the reference genome

| Sample name | Total reads | Total mapped | Multiple mapped | Uniquely mapped |
| --- | --- | --- | --- | --- |
| CK-1 | 43285066 | 41605830(96.12%) | 40034820(92.49%) | 1571010(3.63%) |
| CK-2 | 45889806 | 43960573(95.80%) | 42260939(92.09%) | 1699634(3.70%) |
| CK-3 | 43410908 | 41666884(95.98%) | 40202881(92.61%) | 1464003(3.37%) |
| HY5-1 | 42340760 | 40062013(94.62%) | 37331930(88.17%) | 2730083(6.45%) |
| HY5-2 | 44034966 | 41621457(94.52%) | 38735992(87.97%) | 2885465(6.55%) |
| HY5-3 | 44636280 | 42677423(95.61%) | 40141360(89.93%) | 2536063(5.68%) |

Table S3 Primer sequences

| **Gene name** | **Primer sequence** | **Note** |
| --- | --- | --- |
| *GAPDH* | F: CTGCCAAGGCTGTAGGTAAGG  R: TCAGATTCCTCCTTGATGGCG | qRT-PCR |
| *GbHY5* | F: ATGAAAAGAGTGGACGGTTCT | Gene |
|  | R: TTAGGCATCAGCAGAAAATATTAC | cloning |
| *GbHY5* | F: GCTCTAGATGAAAAGAGTGGACGGTTCT | construction |
|  | R: CCCTCGAGTTAGGCATCAGCAGAAAATATTAC | vector |
| *GbHY5* | F: ACAACTGCGGCTGTGCTGATC  R: TCCAGGTGGAGCAGGTGAAGTAG | qRT-PCR |
| *GbMYB1* | F: AGCAGATGAACGCATTAAGG  R: ATGGGTCGTTCTGCTTGTTG | qRT-PCR |
| *GbFLS* | F: GTTACCCACCGCAGAATGG  R: CACGTAGATCGATGACGGG | qRT-PCR |
| *proGbFLS* | F: CATACACACAGCGTAATGACAAAAT  R: GCTGGCAAGCTTTCTCTACAAA | promoter cloning |
| *GbMYB1* | F: ATGAAGAGTGATAGCAAGGGCAA  R: ACTATTTTGTCTCTTTGCCTGTTCAC | Gene cloning |
| *GbFLS* | F: gaccccgggggtaccggatccATGGCGGCCGAAGCAGCG  R: tttacccatgaattcggatccTTGGCTTAACTTGTTGATTTTGCG | vector construction |
| *proGbFLS* | F: gaccccgggggtaccggatccATGTCTTCTCTGGAGTTCAATATGCC  R: tttacccatgaattcggatccAAGCAGTGCAGCAATTGAGCC | vector construction |
| *GbMYB1* | F: gaccccgggggtaccggatccATGAAGAGTGATAGCAAGGGCAA  R: tttacccatgaattcggatccACTATTTTGTCTCTTTGCCTGTTCAC | vector construction |
| PGBKT7-  GbHY5 | F: aggccgaattcccggggatccATGAAAAGAGTGGACGGTTCTAAAG  R: ccgctgcaggtcgacggatccTTAGGCATCAGCAGAAAATATTACCC | vector construction |
| PGADT7-  GbMYB1 | F: gccatggaggccagtgaattcATGAAGAGTGATAGCAAGGGCAA  R: ccgtatcgatgcccacccgggACTATTTTGTCTCTTTGCCTGTTCAC | vector construction |
